# Clinical Statistical Study on the Prevalence of Carious Lesions in First Permanent Molars

**DOI:** 10.3390/jcm14030669

**Published:** 2025-01-21

**Authors:** Mihaela Jana Țuculină, Andreea Mariana Bănățeanu, Adela Nicoleta Staicu, Alexandru Dan Popescu, Jaqueline Abdul-Razzak, Ionela Teodora Dascălu, Cristian Levente Giuroiu, Veronica Mercuț, Monica Scrieciu, Oana Amza, Mihaela Ionescu

**Affiliations:** 1 Department of Endodontics, Faculty of Dentistry, University of Medicine and Pharmacy of Craiova, 200349 Craiova, Romania; mtuculina@yahoo.com (M.J.Ț.); alexandrudanpopescu20@gmail.com (A.D.P.); 2 Department of Prosthodontics, Faculty of Dental Medicine, University Titu Maiorescu of Bucharest, 67A Gheorghe Petrascu Str., 031593 Bucharest, Romania; andreea.banateanu@prof.utm.ro; 3 Doctoral School, University of Medicine and Pharmacy of Craiova, 200349 Craiova, Romania; jaquelineabdulrazzak90@gmail.com; 4 Department of Infant Care–Pediatrics–Neonatology, University of Medicine and Pharmacy of Craiova, 200349 Craiova, Romania; 5 Department of Orthodontics, Faculty of Dental Medicine, University of Medicine and Pharmacy of Craiova, 200349 Craiova, Romania; marceldascalu@yahoo.com; 6 Department of Endodontics, Faculty of Dentistry, University of Medicine and Pharmacy Grigore T Popa Iasi, 700115 Iasi, Romania; 7 Department of Prosthodontics, Faculty of Dental Medicine, University of Medicine and Pharmacy of Craiova, 200349 Craiova, Romania; veronica.mercut@umfcv.ro (V.M.); monica.scrieciu@umfcv.ro (M.S.); 8 Department of Endodontics, Faculty of Dentistry, University of Medicine and Pharmacy Carol Davila Bucharest, 050474 Bucharest, Romania; oana_amza@yahoo.com; 9 Department of Medical Informatics and Biostatistics, Faculty of Dental Medicine, University of Medicine and Pharmacy of Craiova, 200349 Craiova, Romania; mihaela.ionescu@umfcv.ro

**Keywords:** dental caries, first permanent molar, the frequency of snacks, ICDAS

## Abstract

**Background:** Dental caries remains one of the most widespread chronic diseases that also affects first permanent molars (FPMs). In this study, we analyzed the prevalence of carious lesions using a standardized evaluation system, while following the analysis of the influence of favoring factors represented by the type and frequency of snacks and the frequency of tooth brushing. **Method:** A clinical–statistical study was carried out on a group of 311 children from both urban and rural environments, aged between 6 and 19 years old. As a diagnostic system and assessment of the depth of carious processes, we used the ICDAS system. The following parameters were recorded: odontal status of the 4 FPMs, age, gender, residence, frequency of dental brushing, and frequency of between-meal snacks and their type. The ICDAS system was used as a diagnostic system and assessment of the depth of carious processes. The data were statistically analyzed with SPSS, using the Kruskal–Wallis H and Chi-Square tests. **Results:** The occlusal surface was the most interesting in the presence of odontal lesions. Sweet snacks were the most frequent snacks consumed by the subjects. The age group of 13–19 years presented fewer healthy FPMs, compared to the age group of 6–12 years. The higher involvement of older subjects was found for all four molars, both in the case of identified carious lesions and the presence of root debris and edentations. Brushing twice a day was more frequent in the 6–12 age group. In analyzing the status of the first molars in relation to the gender of the subjects, in the present study, no statistically significant differences were recorded between the status of the molars relative to gender, except for molar 1.6 (*p* > 0.05). Regarding the other types of snacks, children from rural areas consume more fruits than those from urban areas. **Conclusions:** The frequency of tooth brushing and the type and frequency of snacks between meals influence the prevalence of carious lesions at the level of the first permanent molars.

## 1. Introduction

Dental caries is a chronic disease that is widespread worldwide [1]. According to the WHO, dental caries affects between 60 and 90% of schoolchildren globally, particularly in developing countries [2]. Tooth decay is a multifactorial disease resulting from the complex interaction of three key factors: microbiological (biofilm), nutrient substrate (fermentable carbohydrates in the diet), and host susceptibility (teeth and saliva) [3].

Although the etiology of caries is well known, more research is needed to determine the groups of patients at highest risk of caries and to plan appropriate preventive treatment strategies in accordance with the etiologic factors. This would ensure more effective management of the condition while optimizing patient monitoring [4].

The WHO recommends the implementation of regular national oral health surveys, including the monitoring of ten oral health parameters in precisely defined age groups [5].

The first permanent molars [FPM] plays an essential role in masticatory function, helping to establish and maintain occlusion. The tooth also bears the maximum occlusal load, and its edentulization decreases masticatory efficiency by up to 50% [6]. At the same time, it influences the maintenance of the vertical occlusal height and thus the proportions of the facial stages, which define esthetics [7]. Because it has the most voluminous and divergent roots, it is also considered the best source of fixation for tooth movement during orthodontic treatment [8].

Considering the low number of research studies carried out in the Oltenia area on caries in the population, we aimed to conduct a clinical–statistical study in which the prevalence of carious lesions at the level of the FPM was analyzed using the ICDAS diagnostic criteria. The ICDAS is a complex, logical, evidence-based system for caries detection and classification in dental education, clinical practice, epidemiologic research, and publications [9]. The ICDAS has been used in many studies, allowing for the identification of cavitary and non-cavitary carious lesions based on clinical visual appearance [10].

The advantage of the ICDAS index is that it can distinguish between the stages of caries progression in hard dental structures [11,12].

It is well known that the factors listed above play an important role in the appearance and rapidity of the evolution of dental caries, a fact highlighted in several studies [13,14].

Furthermore, the present study aimed to describe the prevalence of caries at the FPM level, in correlation with the favoring and predisposing factors such as the frequency of dental brushing, type of snacks between meals, and their frequency.

## 2. Materials and Methods

The study group consisted of 311 pupils from both rural and urban areas, aged between 6 and 19 years. The sample size was computed using G*Power 3.1.9.7, Heinrich Heine University Düsseldorf, Germany, considering a significance level α of 0.05, a power 1 − β equal to 0.95, and a medium effect size value of 0.275 (with an awareness of practical significance), resulting in a study lot of a minimum of 262 participants. The children were divided into two age groups: group A of 6–12-year-olds and group B of 13–19-year-olds. Clinical examinations were performed between 1 February 2021 and 15 December 2021, by a single examiner, a Ph.D. holder, at a private practice.

As study inclusion criteria, we established the following:

- All two lower first molars were erupted.
- The subjects examined were within the age range of 6–19 years.
- The subjects examined were from the Olt country.

Exclusion criteria were as follows:

- Presence of a general chronic disease.
- Presence of other changes in the odonto-parodontal sphere (periodontal diseases, dental dystrophies, dental trauma, and dental dyschromias following fluoride and antibiotic administration).

For each pupil, an electronic observation form was prepared, in which, in addition to the odontal status of the 4 FPMs, data on age, gender, residence, frequency of dental brushing, frequency of between-meal snacks, and their type were collected.

The present study was approved by the Ethics Commission of the University of Medicine and Pharmacy of Craiova, Romania (08/20.01.2021), in accordance with all ethical principles for studies on human subjects. In addition, all participants and their legal representatives expressed their informed consent to participate in this study.

The clinical examination was performed by inspection with a dental mirror and palpation with a dental probe, systematically, under conditions of good oral hygiene, with the dental surfaces dry and well lighted, starting with the first molar in quadrant 1 and ending with the FPM in quadrant 4. The molar numbering was based on the FDI coding system (3.6—left mandibular FPM; 4.6—right mandibular FPM). The diagnosis of caries was based on the ICDAS criteria [10], as shown in Table 1.

According to the ICDAS criteria, we followed not only the presence of carious lesions but also their localization and evolution on the five molar surfaces. Thus, an ICDAS index was assigned to each molar surface.

Molars that showed an ICDAS index of 0 (zero) on all five tooth surfaces were considered to have a caries status of 0, i.e., sound teeth. Molars with even one non-zero ICDAS surface were considered to have caries status.

The status of root remnant was given to the molar in which the supragingival coronal tooth structure was not present, as it had extensive coronal destruction because of the evolution of dental caries. The status of absent tooth was given to the molar that was not present on the arch or intraosseous, following radiological analysis.

A partially treated tooth was considered as the tooth in which an untreated carious lesion was identified on one tooth surface and a treated and filled carious lesion on another tooth surface.

The initial centralization of the primary data obtained in the study was performed using Microsoft Excel 365 (San Francisco, CA, USA), and the data were divided into subgroups according to the defined variables. Statistical processing was performed using IBM Statistical Package for the Social Sciences (SPSS), version 26.0 (IBM Corp., New York, NY, USA). For the evaluation of intra-examiner calibration, the degree of agreement, expressed by a kappa value, was determined using 20 FPMs classified twice according to the ICDAS system. The strength of agreement for kappa values was conventionally interpreted as follows: less than 0.20 represents a poor concordance; values between 0.21 and 0.40 indicate a fair concordance; values between 0.41 and 0.60 indicate a moderate concordance; values between 0.61 and 0.80 indicate a good concordance; and values between 0.81 and 1.00 indicate a very good concordance.

Criteria for assessing the frequency of dental brushing: In order to assess the frequency of dental brushing, we questioned the patient during the anamnesis and recorded in the patient’s chart their answer regarding this aspect: dental brushing once a day, twice a day, once every 3–4 days, or once every 7 days.

Criteria for assessing the frequency and type of snacks between meals: To assess the frequency of between-meal snacking, we similarly recorded in the patient’s chart one of the following response options obtained during the anamnesis: three times a day, twice a day, once a day, occasionally, or rarely/never. At the same time, we collected information on the type of these foods, filling in one of the following answer options: sweets, salty snacks, fruits/vegetables, or combined.

This study comprised a single continuous variable (children’s age), for which normality and homogeneity were analyzed using Shapiro–Wilk tests for a normal distribution and Levene’s test for homogeneity of variances. Subsequently, for a more eloquent statistical analysis, the pupils participating in the study were divided into two age groups, 6–12-year-olds and 13–19-year-olds.

The choice of these age groups was based on the paternity of dental eruption, i.e., at 6 years of age, the FPM erupts, and at 12 years of age, the transition from mixed dentition to permanent dentition takes place, at which point only permanent teeth are present in the oral cavity.

Continuous variables were expressed as average ± standard deviation (SD); nominal data (demographics, presence of caries, odontal status) and ordinal data (ICDAS index) were expressed as absolute and relative frequencies (%).

For continuous variables, normality was assessed using the Kolmogorov–Smirnov/Shapiro–Wilk test, and correlations were established using the Mann–Whitney test (for data with no normal distribution). For nominal variables, the associations were assessed using the Chi-Square test, and for ordinal variables, the non-parametric Kruskal–Wallis test was used. The results were considered statistically significant according to the α threshold defined as a percentage and for the following *p* values: α = 5% and *p* < 0.05 were considered for a significant result, with a 95% confidence interval (CI).

## 3. Results

The study group comprised 311 children, aged between 6 and 19, with an average of 10.23 ± 2.31 years, divided into two age groups. The distribution of the study lot according to gender, age group, and residence is shown in Table 2.

The distribution of children in the two age groups, relative to gender and residence, is shown in Table 3.

Within the study group, the children in the two age groups were evenly distributed with respect to gender and residence, with no significant differences (*p* > 0.05, Table 4).

Because the ages of the students were not normally distributed, a Mann–Whitney U test was carried out to determine if there were any differences between the ages of children of different gender. The age distributions for girls and boys were similar. The median age for girls was 11.0 years, and for boys, it was 10.5 years; thus, no statistically significant differences between girls and boys were identified: U = 11,875.00; z = −0.144, *p* = 0.885. Similarly, the median age of children from rural and urban residence was 11.00 years, with no statistically significant differences between the two residence types: U = 12,983.00; z = 1.223, *p* = 0.221.

### 3.1. The Odontal Status of Molars Relative to the Age Group Distribution

The majority of the children in both age groups had molar 1.6 indemnity (85.1% for the first group, and 71.4% for the second group). Both age groups had root debris of molar 1.6 (0.8% for the 6–12 age group, compared to 3.6% for the 13–19 age group) and untreated caries of this molar (12.9% for the 6–12 age group, compared to 16.1% for the 13–19 age group). Overall, the differences in molar 1.6 status between the two groups were statistically significant (*p* = 0.007).

For molar 2.6, the differences in the status of the indemnity were greater between the two age groups, with 87.5% for younger children and 64.3% for older children. A quarter of the children aged 13 to 19 years had molar 2.6 with untreated carious lesions, compared with only 11% of younger children. There were no children with root debris. The differences between the two groups relative to molar 2.6 status were statistically significant (*p* < 0.0005).

Regarding molar 3.6, only 69.4% of the children aged 6–12 years had molar 3.6 indemnity compared to only 41.1% of the children aged 13–19 years.

For both age groups, the percentage of children with untreated carious lesions in this molar increased: 23.1% for younger children and 41.1% for older children. The percentage of root debris at this level also increased, compared to the other two molars previously presented (1.2% for the 6–12 age group and 1.8% for the 13–19 age group). Also, for molar 3.6, the differences between the two groups were statistically significant (*p* = 0.005, Table 4).

Molar 4.6 was the most affected molar in the whole study group. Only 67.8% of the children in the 6–12 age group had this molar indemnity, and only 32.1% of the children in the 13–19 age group had this status. Again, the percentages of untreated caries at this level increased, with 26.3% for the first age group and 53.6% for the second age group. The differences between the two age groups were statistically significant relative to molar status 4.6 (*p* < 0.0005, Table 4).

### 3.2. The Distribution of the Study Group Relative to FPM, Gender, and Residence

Statistically significant differences in FPM status in relation to gender were found only in molar 1.6 (*p* = 0.047, Table 5). Regarding the root rest status of molar 1.6, it was observed in 75% of male children and only 25% of female children. The presence of decayed molar 1.6 was found predominantly in girls (10.3% of the total number of children included in the study group), while in boys, only 5.1% of the total number of children participating in the study were found. Following the examination of the carious lesions present on molar 1.6, the untreated caries status is predominant in 13.5% of the entire group of participants.

The odontal status of molars 2.6, 3.6, and 4.6 did not show statistically significant differences in relation to the gender of the study participants, with both girls and boys having a similar status (*p* > 0.05, Table 5).

Analysis of the odontal status of molars 1.6, 2.6, 3.6, and 4.6 with respect to the participants’ residence showed no statistically significant differences between these two categories (*p* > 0.05, Table 5).

### 3.3. Distribution of Carious Lesions by Localization (Surfaces) and Diagnosis (ICDAS Indices)

With respect to intra-examiner reliability, the kappa value for the ICDAS index for the examiner performing this study was 0.921, proving a very good concordance level.

The occlusal surface of molar 1.6 was more affected in the 13–19 age group, with 17.86% of teeth with carious lesions present (10 teeth out of a total of 56), compared to 10.98% of teeth with carious lesions present for the other age group (28 teeth out of a total of 255), with the differences being statistically significant (*p* = 0.032). On the other hand, there were no statistically significant differences in the ICDAS index between the two age groups (*p* = 0.700). On the mesial, buccal, distal, and oral surfaces, no statistically significant differences were found between the two age groups, neither in the level of caries present on these surfaces of molar 1.6 nor in the ICDAS index values (*p* > 0.05).

Regarding the presence of caries on the affected surfaces of molar 1.6 in relation to the gender of the study participants, both girls and boys show similar values. The only statistically significant difference was in the ICDAS index for the occlusal face (*p* = 0.046), as only 84.3% of the girls had an ICDAS index of 0 (144 girls out of 171), compared with 92.1% of the boys (129 boys out of 140). For the other values of the index, from 2 to 5, this gender imbalance was also maintained, with the percentage of girls always being at least 70% of the total number of children with the respective index.

The environment of origin does not seem to influence the presence of caries on the faces of molar 1.6, nor the values of the ICDAS index, with the results of the statistical tests not being significant (*p* > 0.05).

The occlusal surface of molar 2.6 was more affected for the 13–19 age group, with 23.64% of teeth with carious lesions present (13 teeth out of 56 total) compared to 10.16% of teeth with carious lesions present for the other age group (26 teeth out of 255 total), with the differences being statistically significant (*p* < 0.0005). Significant differences between the two age groups also occurred for the ICDAS index, where 12.50% of the surfaces in the 13–19 age group had an index of 4 (7 teeth out of a total of 56), compared with only 2.75% for children younger than 13 years (7 teeth out of a total of 255). Differences were also present for an ICDAS index of 0, with 89.80% for the 6–12 age group (229 teeth out of a total of 255) versus 76.79% for the 13–19-uear-old group (43 teeth out of a total of 56). At the ICDAS index level, the differences between groups were statistically significant (*p* = 0.015).

The mesial surface area was affected to a similar extent by the presence of caries, with statistically significant differences between the two groups (*p* < 0.0005): 3.14% caries present in the 6–12 age group (8 teeth out of 255), compared to 23.21% for the 13–19 age group (13 teeth out of 56). The ICDAS index also showed statistically significant differences (*p* < 0.0005), with the 13–19 age group having only 76.79% of surfaces with an index of 0 (43 teeth out of a total of 56), versus 96.86% for the 6–12 age group (247 teeth out of a total of 255).

The vestibular surface did not show statistically significant differences between the two groups, neither in the presence of caries nor in the values of the two ICDAS indices. There were no statistically significant differences between the two age groups on the oral surface of molar 2.6, either in terms of caries present on this surface or in the ICDAS two index values (*p* > 0.05).

The distal surface showed statistically significant differences between the two age groups (*p* = 0.015), only in terms of the presence of caries, as follows: 14.29% of the surfaces in the 13–19 age group (8 teeth out of 56) were affected by caries, compared with only 5.88% of the surfaces in the 6–12 age group (15 teeth out of 255). The ICDAS index had similar values for the two groups (*p* > 0.05).

Regarding the presence of caries on the surfaces of molar 2.6 in relation to the gender of the study participants, no statistically significant differences were found for any of them. However, for the ICDAS values corresponding to the distal surface, there were statistically significant differences in relation to the gender of the children participating in the study (*p* = 0.022), with the female gender being more affected. An ICDAS index of 2 on the distal surface was defined mainly for boys (60% of the total number of surfaces with that index), compared to 40% for girls. Similarly, an ICDAS index of 3 on the distal surface was defined mainly for females (66.7% of the total number of areas with the respective index), compared to 33.3% for males. On the other hand, an ICDAS index of 5 on the distal surface was identified only in girls.

The analysis carried out in relation to the environment of origin did not reveal statistically significant differences in the ICDAS index values for the faces of molar 2.6. Only the presence of caries on the mesial face of this molar was identified in rural children (66.7%, 14 children out of 21 children with caries on this surface) compared to only 33.3% in urban children (7 children).

For molar 3.6, all occlusal surfaces showed statistically significant differences between the two age groups (*p* < 0.0005). However, the involvement was also high in the 6–12 age group, with 21.96% of the surfaces affected by the presence of caries (56 teeth out of 255 total), reaching 33.93% for the surfaces in the 13–19 age group (19 teeth out of 56 total). Significant differences between the two age groups also appeared in the ICDAS index (*p* = 0.015), where 79.22% of the occlusal surfaces in the 6–12 age group had an index of 0 (202 surfaces out of 255 total), compared to the other group, where only 60.71% of the surfaces had this value (34 surfaces out of 56 total). There were also significant differences for the ICDAS value of 3, with 19.64% affected for the 13–19 age group (11 surfaces out of the total of 56), compared to 5.10% for the 6–12 age group (13 surfaces out of the total of 255). The vestibular surface was less affected than the occlusal surface for molar 3.6, with 9.80% caries present in the 6–12 age group (25 surfaces out of 255), and 17.86% caries present in the 13–19 age group (10 surfaces out of 56), with statistically significant differences (*p* = 0.034). On the other hand, the ICDAS index had similar values for both groups (*p* > 0.05).

On the mesial, distal, and oral surfaces, there were no statistically significant differences between the two age groups, neither in the level of caries present on these surfaces of molar 3.6 nor in the ICDAS index values (*p* > 0.05).

Regarding the status of the affected surfaces of molar 3.6 in relation to the gender of the study participants, only the presence of caries on the distal surface was significantly different, with caries being identified predominantly in girls (74.2%) compared to 25.8% in boys.

The analysis carried out in relation to residence did not reveal statistically significant differences in the presence of caries or ICDAS index values for molar 3.6 surfaces.

Molar 4.6 showed the highest number of occlusal surfaces affected by caries of the entire study group, with 9.80% of caries present for children younger than 12 years of age (25 teeth out of a total of 255), and 25.00% caries present for children 13 years of age and older (14 teeth out of a total of 56). The differences were statistically significant between the two age groups (*p* < 0.0005).

The differences for the ICDAS index showed a statistical *p*-value at the borderline of significance (*p* = 0.052), with 89.80% occlusal surfaces with an ICDAS index of 0 for the 6–12 age group (229 teeth), compared to 76.79% occlusal surfaces with an ICDAS index of 0 for the other age group (43 teeth).

There were also statistically significant differences in the mesial surface area, both in terms of the presence of caries and the ICDAS index values (*p* = 0.006). Thus, molars 4.6 in the 6–12 age group had 3.14% surfaces affected by the presence of dental caries (8 teeth out of a total of 255), compared to 23.21% surfaces with carious lesions in the 13–19 age group (13 teeth out of a total of 56).

Also, there were 96.86% surfaces with an ICDAS index of 0 for the toddler group (247 surfaces out of the total of 255), compared to only 76.79% surfaces with an ICDAS index of 0 for the 13–19 age group (43 surfaces out of the total of 56).

As for the ICDAS index of 3, a percentage of 1.18% was obtained for children aged 6–12 years (2 teeth out of a total of 255), versus 12.50% for children aged 13–19 years (7 teeth).

For the distal surfaces of molars 4.6 of the two groups, there were no statistically significant values relative to the presence of dental caries on these surfaces or to the ICDAS index values.

The buccal and oral surfaces did not show statistically significant differences between the two age groups for any of the parameters studied at this level.

Molar 4.6 showed no change in surfaces in relation to the gender of the study participants or their residence. Likewise, no changes were found in any of the ICDAS surface area indices. The detailed situation is presented in Table 6.

### 3.4. Frequency of Dental Brushing

There is a statistically significant difference between the two age groups in terms of frequency of tooth brushing (*p* = 0.002, Table 7). The majority of the children analyzed in terms of frequency of tooth brushing are in the 6–12 age group (81.99%), while only 18.01% of them are in the 13–19 age group. For the category of 6–12-year-olds, 63.5% brush their teeth twice a day; 24.7%—once a day; 10.2%—once/3–4 days; and 1.6%—once/7 days. The same trend can be observed for the 13–19 age group, with 37.5% brushing their teeth twice a day; 35.7%—once a day; 21.4%—once/3–4 days; and 5.4%—once/7 days.

The highest percentage of children, irrespective of age group, was, however, of those who brush their teeth twice a day. The frequency distribution of dental brushing is shown in Table 7.

#### Odontal Status of Molars Relative to the Frequency of Dental Brushing

In analyzing the data, significant differences can be observed between the status of molar 1.6 relative to the frequency of dental brushing (*p* = 0.0001, Table 8). A large percentage of the children, however, have molar 1.6 untreated, irrespective of dental brushing frequency (39.87%), followed by 13.50% of the children with untreated caries.

The influence of the frequency of dental brushing on the status of molar 1.6 is best observed when analyzing the category of children with untreated caries, where a lower percentage of untreated caries is found in the category of children who brush their teeth twice/day (11.9% of the category), followed by 35.7% children with caries whose frequency of tooth brushing is once/day, and then by the highest percentage in this category—38.1% children with untreated caries who brush their teeth once every 3–4 days.

The frequency of tooth brushing also influences the status of molar 2.6. Significant differences in molar 2.6 status in relation to the frequency of tooth brushing were also found (*p* = 0.0001, Table 8). 

Children with a dental brushing frequency of once/3–4 days had the highest percentage of untreated M2.6 caries (50% of the category) and also of incorrectly treated M2.6 caries (66.7% of the category).

Molar 3.6 shows significant differences in its status relative to dental brushing frequency. In this case also, a fairly high percentage of children have the molar untreated (64.31% of all cases analyzed), followed by 26.37% of children with untreated caries status for molar 3.6. The status of root rest was found in 66.7% (of the category) in children whose frequency of tooth brushing was 1/3–4 days, followed by 33.3% (of the category) of children who brush their teeth only once/7 days. The other two categories of frequency of tooth brushing are not present in this situation.

Molar 4.6 is again found to be the most affected in the whole study group, with only 191 children presenting the unaffected status, compared to 257 for molar 1.6, 259 for molar 2.6, and 200 for molar 3.6.

However, it is noteworthy that 61.41% of the children whose 4.6 molars were studied, in relation to the frequency of dental brushing, showed the toothless status for this molar. The absent status for molar 4.6 accounted for only 0.32% of the cases, for root debris at M4.6, it was 1.6%, and the decayed status was 36.7%.

The distribution of the cases in relation to the frequency of dental brushing, regarding the untreated caries status, revealed that the most affected were children whose frequency of dental brushing was 1/day (42.3% of the category), followed by children whose frequency of dental brushing was 1/3–4 days (28.9% of the category), and then those whose frequency of dental brushing was 2/day (24.7% of the category). The differences between molar status 4.6 versus dental brushing frequency were statistically significant (*p* = 0.0001, Table 8).

### 3.5. The Type and Frequency of Snacks Between Meals

The influence of the residence of the study participants on the type of snacks served by the study participants is statistically significant (*p* = 0.048). The most common category of snacks is sweets; 65.3% of urban children prefer sweets, and 50.7% rural children prefer sweets. As for the other types of snacks, rural children consume more fruit (19.4%) than urban children (11.4%), and combined snacks are preferred by 20.8% of rural children, while urban children account for 18.0%.

The type of snacks influences the frequency of dental brushing irrespective of the category of the children, which is also reinforced by the coefficient *p* = 0.001. It can be observed that sweets-type snacks are associated with an increase in tooth brushing as follows: frequency—2/day with a percentage of 60.7% (of the total category); frequency—1/day with a percentage of 59.0% (of the total category); frequency—1/3–4 days with a percentage of 50.0% (of the total category); frequency—1/7 days with a percentage of 42.9% (of the total category).

Significant differences are also found between the frequency of snacks relative to the type of snacks (*p* = 0.0001). From the analyzed data, it can be observed that the highest percentage of children consume two snacks per day (39.87% of the total number of children who participated in the study), followed by those who eat only one snack per day (27.65% of the total number of children). At the other end of the spectrum, only 5.79% of children rarely or never snack. Sweet snacks are in the majority, regardless of the frequency of snacking. The types of snacks between meals are depicted in Table 9.

#### 3.5.1. Molar Status in Relation to the Type of Snacks

The analysis of each molar in relation to the type of snacks revealed statistically significant differences.

For molar 1.6, it can be observed that the majority of children have it untreated (82.64% of all children participating in the study), but of the remaining categories, the majority have the molar 1.6 decayed and untreated (77.78% of the children with a status other than untreated, for M1.6). There were significant differences in the status of molar 1.6 relative to the type of snacks (*p* = 0.043). Sweet snacks predominate, regardless of the molar 1.6 status, followed by combined snacks. Interestingly, study participants with affected molar 1.6 (root remnant, untreated caries, correctly treated caries, or incorrectly treated caries) reported only eating sweets or combined snacks.

For molar 2.6, the following situation was found: the majority of children presented with molar 2.6 free (83.28%), followed by those with untreated caries (13.50%). Significant differences in the status of molar 2.6 in relation to the type of taste were also found for this molar (*p* = 0.009).

Molar 3.6 is toothless in 64.31% of all children who participated in this study. The affected status of M3.6 is found in the majority of children who consume sweets, with most of them presenting with carious but untreated molar 3.6 (81.16% of the category of children with M3.6 whose status is affected).

Molar 4.6 is found in the indemnity status in 61.41% of the children participating in the study. As for the other M4.6 statuses found, the majority percentage is of subjects with the untreated caries status (80.83% of the category of children with something other than an untreated status for M4.6). Also, in M4.6, the status is affected, in relation to the type of snacks, in those who consume sweets (66.67% of the total of the category presenting a status other than indignant for M4.6), followed by those who consume combined snacks. The detailed situation is presented in Table 10.

#### 3.5.2. Surface Status—ICDAS Index

Molar 1.6. The surfaces affected in terms of the relationship between surface status for molar M1.6 relative to the type of snacking are occlusal (*p* = 0.001), mesial (*p* = 0.02), and oral (*p* = 0.034). However, the type of snacking does not introduce changes in the vestibular and distal surfaces (*p* > 0.05). The only unmodified ICDAS indicator is the vestibular surface, with the rest of the ICDAS indicators for the others being modified, as previously stated.

Molar 2.6. With regard to the status of the affected surfaces of molar 2.6 in relation to the type of taste, statistically significant differences were found for the occlusal (*p* = 0.0001), distal (*p* = 0.048), and oral (*p* = 0.016) surfaces. Changes were also found in the ICDAS index of occlusal and distal surfaces.

The occlusal surface of molar 2.6 is affected in relation to the type of bite, with statistically significant differences (*p* = 0.0001). Significant differences in relation to the type of bite also appear in the ICDAS index (*p* = 0.002).

The medial surface area is also affected, with statistically significant differences between snack types (*p* = 0.034). In contrast, there are no statistically significant differences between snack types for any of the ICDAS indices.

The vestibular surface does not show statistically significant differences in relation to the type of snacks, nor in relation to the ICDAS index values.

The distal surface of molar 2.6 is affected in relation to the type of snacks, with statistically significant differences (*p* = 0.048). Significant differences in relation to the type of snacking also appear for the ICDAS index (*p* = 0.034). The oral surface area of molar 2.6 is affected in relation to the type of snacks, with statistically significant differences (*p* = 0.016). In contrast, ICDAS values do not present statistically significant differences in relation to the type of snacks (*p* > 0.05).

Molar 3.6. The occlusal surface of molar 3.6 is affected in relation to the type of snack, with statistically significant differences (*p* = 0.0001). Significant differences in relation to the type of snack also appear for the ICDAS index (*p* = 0.001). The mesial surface is also affected, with statistically significant differences between the type of snack (*p* = 0.018), but for none of the ICDAS index values are there statistically significant differences in relation to the type of snack.

The vestibular surface of molar 3.6 is affected in relation to the type of snacks, with statistically significant differences (*p* = 0.040). Significant differences in relation to the type of snacking also appear for the ICDAS index (*p* = 0.038). The distal surface of molar 3.6 is affected in relation to the type of snacking, with statistically significant differences (*p* = 0.029). The oral surface does not show statistically significant differences in relation to the type of snack, nor in relation to the ICDAS index values.

Molar 4.6. In molar 4.6, the occlusal surface is affected in relation to the type of snacks, with statistically significant differences (*p* = 0.0001). Significant differences in relation to the type of snacks also appear in the ICDAS index (*p* = 0.0001).

The medial surface area is also affected, with statistically significant differences between snack types (*p* = 0.005). Significant differences in relation to snack type also appear for the ICDAS index (*p* = 0.021). The vestibular surface of molar 4.6 is affected in relation to the type of snack, with statistically significant differences (*p* = 0.005). Significant differences in relation to the type of snacking also appear in the ICDAS index (*p* = 0.005). The distal surface of molar 4.6 is affected in relation to the type of snacking, with statistically significant differences (*p* = 0.013). Significant differences in relation to the type of snacking also appear in the ICDAS index (*p* = 0.021). The oral surface does not show statistically significant differences in relation to the type of snacks, nor in relation to the ICDAS index values. The detailed situation is presented in Table 11.

### 3.6. Analysis of Frequency of Snacks

The most frequent practice in terms of the number of snacks/day is two snacks per day, for both age groups, with 36.5% of children in the 6–12 age group having two snacks per day and 55.4% in the 13–19 age group. However, there are differences between the age groups, and these are statistically significant (*p* = 0.002), with younger children having a higher percentage of occasional or no snacks.

Dental brushing frequency is influenced by snack frequency, with statistically significant differences (*p* = 0.001). Snack type also shows significant differences in snack frequency (*p* = 0.001). Sweet-type snacks predominate, regardless of snack frequency, followed by combined snacks.

Gender and environment did not show statistically significant differences in relation to snack frequency (*p* > 0.05) as shown in Table 12.

#### 3.6.1. Molar Status in Relation to the Frequency of Snacks

The majority of the children have molar 1.6 free, irrespective of the frequency of snacking (82.6%). Only a very small percentage of children have molar 1.6 absent (0.3%), 1.3% have root debris, 13.5% have untreated caries, 0.6% have correctly treated caries, and 1.6% have incorrectly treated caries. Overall, the differences between the six categories for the status of molar 1.6 were not statistically significant (*p* = 0.078).

For molar 2.6, the differences relative to the indemnity status are greater for those with a snack frequency of two/day or one/day, with 35.5% for those with two snacks and 31.7% for those with one snack, respectively. The overall percentage regardless of snack frequency is 83% for children whose status for M2.6 is indemnity. A percentage of 16.4% of the subjects present one of the caries forms (untreated caries—13.5%; correctly treated caries—1.9%; incorrectly treated caries—1.0%). Regarding the influence of the frequency of snacks per day, it is observed that the highest percentage of children with untreated caries on molar 2.6, is that of those who consume two snacks per day (57.1%), followed by the percentage of those who consume three snacks/day (19.0%), with the other categories showing lower percentages relatively close in value. Overall, the differences in the categories of the molar 2.6 status relative to snack frequency were statistically significant (*p* = 0.010).

As for molar 3.6, 64.3% of the children studied have molar 3.6 indemnity, regardless of snack frequency. The absent status for molar 3.6 presents only in 1.3% of the cases; this was reported for the category of two snacks/day. Root remnants in molar 3.6 were found in the category of two snacks/day (1.6% of all participants) and one snack/day (0.3%). The category of molar 3.6 showing caries has a percentage of 32.5 of the total participants, of which untreated caries predominates (26.4%), followed by correctly treated caries (2.9%), and incorrectly treated caries in 3.2% of the cases where molar 3.6 shows caries status. Also, for molar 3.6, the differences between molar status versus the frequency of snacking were statistically significant (*p* < 0.005).

Molar 4.6 again proves to be the most affected molar in the whole study group, with only 191 children showing the toothless status, compared to 257 for molar 1.6, 259 for molar 2.6, and 200 for molar 3.6. However, it is noteworthy that 61.4% of the children whose molar 4.6 was studied, in relation to the frequency of snacking, showed the toothless status for this molar. The absent status for molar 4.6 accounted for only 0.3% of the total cases, for the root remnant at M4.6, it was 1.6%, and the decayed status was 36.7%. The distribution of the cases in relation to the number of snacks per day, concerning the caries status, revealed that the most affected were children who had two snacks/day (20.9%), followed by children who had one snack/day (6.3%), then three snacks/day (5.1%), occasionally (3.5%), and rarely/never (0.6). The differences between the molar 4.6 status versus snack frequency were statistically significant (*p* < 0.005). The detailed situation is presented in Table 13.

#### 3.6.2. Surface Status—Indices

From the data analyzed regarding molar 1.6, it can be observed that the frequency of snacking has an influence, so that the occlusal surface (*p* = 0.01) and the medial surface (*p* = 0.032) are affected, but without significant influence on the ICDAS index. On the mesial, buccal, distal, and oral surfaces, there are no statistically significant differences in the frequency of taste, neither in the level of caries present on these surfaces of molar 1.6 nor in the ICDAS index values (*p* > 0.05).

The condition of molar 2.6 shows, in some respects, an influence on the frequency of snacking. The molar has all surfaces affected. It was also found that the totality of the surfaces relative to the ICDAS index values are affected. Molar 3.6 shows changes in the occlusal, mesial, and distal surfaces in relation to the frequency of snacking. The occlusal, mesial, and distal surfaces were also found to be affected in relation to the ICDAS index values. The vestibular surface showed statistically significant differences in snack frequency, even in terms of the ICDAS index. On the occlusal, mesial, and distal surfaces, there are no statistically significant differences in the frequency of snacking, nor in the level of caries present on the surfaces of molar 3.6, relative to the ICDAS index values. As for the oral surface of molar 3.6, there are no statistically significant differences between the surface condition relative to the frequency of snacking, but neither for the ICDAS index values.

Molar 4.6 shows changes in the occlusal, mesial, buccal, and distal surfaces in relation to snack frequency. It was also found that the same surfaces were also influenced in relation to the ICDAS index values. On the occlusal, mesial, vestibular, and distal surfaces, there were no statistically significant differences in snack frequency. As for the oral surface of molar 4.6, there were no statistically significant differences between the surface conditions relative to the frequency of snacking, but neither for the ICDAS index values. The detailed situation is presented in Table 11.

#### 3.6.3. Distribution of Carious Lesions According to Localization (Surfaces) and Diagnosis (ICDAS Index)—Frequency of Dental Brushing

Statistically significant differences were found in the status of the affected surfaces of molar 1.6 in relation to the frequency of dental brushing, all of which showed dental caries. Semi-significant changes were also found in the ICDAS index of all surfaces.

The occlusal surface of molar 1.6 is affected in relation to dental brushing frequency, with statistically significant differences (*p* = 0.0001), with children who brushed once a day or less frequently showing the most caries. Significant differences in relation to tooth brushing frequency also appear for the ICDAS index (*p* = 0.0001). Children who brush their teeth most often have the lowest index values. The mesial surface is also affected, with statistically significant differences between the status of the mesial surface in relation to the frequency of tooth brushing (*p* < 0.0005). Significant differences in relation to the frequency of dental brushing also occur for the ICDAS index (*p* < 0.0005).

The vestibular surface is affected, with statistically significant differences between the vestibular surface status and dental brushing frequency (*p* < 0.000). Significant differences in relation to the frequency of dental brushing also appear for the ICDAS index (*p* < 0.0005).

The distal surface of molar 1.6 is affected in relation to dental brushing frequency, with statistically significant differences (*p* = 0.0001). Significant differences in relation to the frequency of dental brushing also appear for the ICDAS index (*p* = 0.0001,). The oral surface area of molar 1.6 is affected in relation to dental brushing frequency, with statistically significant differences (*p* = 0.001). Significant differences in relation to dental brushing frequency also appear for the ICDAS index (*p* = 0.002).

With regard to the status of the affected surfaces of molar 2.6 in relation to the frequency of dental brushing, statistically significant differences were found for occlusal (*p* = 0.0001), mesial (*p* = 0.0001), distal (*p* = 0.001), and oral (*p* = 0.001) surfaces. Changes were also found in the ICDAS index of occlusal, mesial, oral, and distal surfaces. The occlusal surface of molar 2.6 is affected in relation to the frequency of dental brushing, with the differences being statistically significant (*p* = 0.0001). Significant differences in relation to the frequency of tooth brushing also appear for the ICDAS index (*p* = 0.0001). The mesial surface is also affected, with statistically significant differences in relation to the frequency of dental brushing (*p* = 0.001), and the same is true for the ICDAS index values. The vestibular surface does not show statistically significant differences with respect to the frequency of tooth brushing, nor with respect to the ICDAS index values (*p* > 0.05).

The distal surface of molar 2.6 is affected in relation to the frequency of dental brushing, with statistically significant differences (*p* < 0.0005). Significant differences in relation to the frequency of dental brushing also appear for the ICDAS index. The oral surface of molar 2.6 is found to be affected in relation to the frequency of tooth brushing, with statistically significant differences. Significant differences in relation to the frequency of tooth brushing also appear in the ICDAS index. The occlusal surface of molar 3.6 is affected in relation to the frequency of dental brushing, with statistically significant differences (*p* = 0.0001). Significant differences in relation to the frequency of tooth brushing also appear for the ICDAS index (*p* < 0.05). The mesial surface is also affected, with statistically significant differences with dental brushing frequency (*p* = 0.041), and statistically significant differences also occur for the ICDAS index (*p* = 0.015). The vestibular surface of molar 3.6 is affected in relation to dental brushing frequency, with statistically significant differences (*p* < 0.0005). Significant differences in relation to the frequency of dental brushing also appear for the ICDAS index (*p* = 0.0001). The distal surface of molar 3.6 is affected in relation to dental brushing frequency, with statistically significant differences (*p* = 0.0001), but also for ICDAS index values. The oral surface shows no statistically significant differences with respect to the frequency of dental brushing, nor with respect to the ICDAS index values.

The occlusal surface of molar 4.6 is affected in relation to the frequency of dental brushing, with statistically significant differences (*p* < 0.05). Significant differences in relation to dental brushing frequency also appear for the ICDAS index (*p* < 0.05). The mesial surface is also affected, with the differences being statistically significant in relation to dental brushing frequency (*p* = 0.0001), and statistically significant differences also appear for the ICDAS index (*p* < 0.05). The vestibular surface of molar 4.6 is affected in relation to dental brushing frequency, with the differences being statistically significant (*p* = 0.03). Significant differences in relation to the frequency of dental brushing also appear for the ICDAS index (*p* = 0.0001). The oral surface does not show statistically significant differences in relation to the frequency of dental brushing, nor in the ICDAS index values.

## 4. Discussion

FPMs play a key role in establishing normal occlusal ratios; therefore, the health of the entire oral cavity depends on their health. In addition to the morphology that favors bacterial biofilm retention and thus caries development, the lack of parental knowledge about the belonging of these teeth to the permanent dentition makes the FPMs the most affected permanent teeth [15,16].

The importance of the carious pathology of FPMs has been clinically known for a long time. Epidemiologic surveys conducted in several countries have shown the extent of this problem worldwide [17,18]. Several studies have shown a high prevalence of caries in the FPM, even shortly after eruption; therefore, the need to apply prevention programs is obvious, especially for the first decade of life. Even in countries where local and general dental caries prevention measures are routinely applied, the occlusal surface of the FPM remains the site of choice for carious processes [6,19]. Also, the degree of damage to the FPM is an indicator of the health of the entire permanent dentition, as significantly increased caries damage to the other permanent teeth has been found when the FPM is affected by carious lesions [20].

### 4.1. Analysis of Results Reported by Age Groups

The results of our study are in agreement with other specialized studies, with the prevalence of carious lesions being increased in the groups of children examined. The results are related to a number of parameters such as age, gender, and residence, as well as the frequency of dental brushing and type and frequency of between-meal snacks. The results of this analysis were compared with those of other relevant national and international studies.

Analyzing the odontal status of FPMs, we found that the 13–19 age group showed fewer FPMs free compared to the 6–12 age group. The higher involvement of the older subjects was found for all four molars, both in identified carious lesions and in the presence of root debris and edentulism. Other studies also estimate that the prevalence of dental caries is higher in older children [21].

In analyzing the surface localization of the identified carious lesions, for all four molars, the occlusal surface was the most concerned for both age groups. Corresponding to the other surfaces, the involvement was much lower, and statistically significant differences between the two age groups were recorded for the mesial and distal surfaces of molars 2.6 and 4.6, and the buccal face of molar 3.6, respectively. In all cases, the 13–19 age group showed higher involvement. One explanation for this phenomenon is that with increasing age, children experience the independence of performing their own oral hygiene, often neglecting the correctness of oral hygiene. Moreover, adolescents are making more choices about their diet, which may include a high consumption of sweet and processed foods. In other words, parents’ control over their child’s oral hygiene and diet is decreasing and the responsibility is largely left to the child, who may not fully realize the importance of oral health [22].

In a study conducted in Romania by Abel Emanuel Moca et al. on a group of children aged 6–14 years, which aimed to find a correlation between age and the evolution of dental caries at the FPM, it was found that there was an association between chronologically younger age and superficial carious lesions. With increasing chronologic age or dental age, an increase in the number of medium and deep carious lesions was also found [23]. This finding is in agreement with the results of our study. The carious lesions diagnosed based on the ICDAS criteria were found to be deeper, showing indices 4 and 5 more frequently for occlusal surfaces in the 13–19 age group. These results were obtained for all the molars examined, except for molar 1.6, which did not differ significantly between the two age groups.

With regard to the degree of the advancement of carious lesions, it is important to note that in younger children, the responsibility for regular check-ups rests entirely with the parents, which leads to a higher rate of referral to dental health services. Thus, primary and secondary preventive measures are more easily implemented during this period [24].

With increasing age, the decision to seek dental services is not only the responsibility of the parents, but also of the adolescent, who must signal the need or desire to see a dentist. According to studies, after the age of adolescence, the main reason why subjects go to the dentist is the onset of pain [25]. In addition to pain, another factor that may lead older children to want to solve their dental problems is the onset of concern about physical appearance and, implicitly, dental esthetics [26]. The lack of regular check-ups may be caused by the onset of anxiety, which is more evident around the age of 15 [27].

The higher caries involvement of older subjects can also be explained by the influence of the favorable factors identified in this study, namely the frequency of tooth brushing and the type and frequency of snacks between meals. Brushing twice a day was more frequent in the 6–12 age group.

According to a study in Cluj, more than a third of teenagers rarely or never brush their teeth in the evening. Their eating habits and irregular dental check-ups have been associated with dental caries [18]. Other comparative studies revealed that Romanian adolescents visit the dentist less often, brush their teeth less often than adolescents in other European Union countries, and consume sugary foods more frequently [28].

In terms of sweets consumption, the American Heart Society recommends that a child’s maximum daily sugar intake should not exceed 6 teaspoons [29].

Moynihan et al. showed that in the field of dental medicine, more important than the amount of sugar ingested is the frequency of intake [28].

A diet that contains vegetables, fruits rich in vitamin C and dairy, provides the body with sufficient calcium, which plays an important role in the prevention of dental caries. In other words, in addition to the frequency of between-meal snacks, the type of snacks is also very important. Snacks represented by fruits and vegetables, thanks to their rich water content [between 75 and 90%], dilute the sugar content they contain and stimulate salivary flow [30].

In terms of the gender distribution of fruit and vegetable consumption, studies show that girls consume more fruit daily than boys [31]. In our study, there were no statistically significant differences in fruit and vegetable consumption in relation to the gender of the subjects; however, the influence of the participants’ residence on the type of snack was statistically significant. The most common snack category is sweets, with a higher percentage of children in urban areas consuming sweets. In terms of other types of snacks, rural children consume more fruit than urban children, and combined snacks are preferred by 20.8% of rural children, while urban children constitute 18.0%.

Studies show a clear tendency of adolescents to skip breakfast, which leads to the consumption of more snacks, predominantly those rich in carbohydrates [32]. This tendency was also observed in our study, with statistically significant differences between age groups (*p* = 0.002), with the 13–19 age group having a lower percentage of frequency of occasional snacking or no snacking.

### 4.2. Analysis of Results Related to the Gender of the Subjects

In analyzing the molar status of the first molars in relation to the gender of the subjects, in the present study, there were no statistically significant differences between the molar status in relation to the gender of the subjects. An exception was molar 1.6, in which carious lesions were twice as common in girls as in boys. However, a three times higher proportion of root debris was noted in boys.

Although there studies have claimed that the female gender is more prone to caries lesions in the FPM due to their earlier eruption and lower salivary secretion [33], the literature is replete with studies supporting a similar caries involvement of both genders [34,35,36]. The influence of gender on the prevalence of dental caries in the FPM is low and inconsistent according to the results of our study.

### 4.3. Analysis of Results by Residence

Although access to prevention and oral care programs in urban areas should theoretically decrease the prevalence of dental caries [37], there are studies that demonstrate the opposite [38].

When comparing the status of the molars analyzed to their residence, no statistically significant differences were found for any molar. These results may also be justified by the finding that the frequency of dental brushing is low and similar for the two residence types.

Statistically significant differences were recorded only for the distal face of molar 3.6 and for the ICDAS values at the occlusal surface of molar 1.6 and the distal surface of molar 2.6. Thus, the results of our study, according to the residence setting, are in agreement with other studies that did not find statistically significant differences in the prevalence of dental caries [17,39].

However, irrespective of the previous parameters analyzed [age, environment, gender], dental brushing frequency, and type and frequency of snacks between meals, the results show that the lower molars are the most affected, and concerning the localization of carious processes, the occlusal face was the most concerned. An explanation for this finding could be the presence of an occlusal retentive morphology and difficult access to perform more effective oral hygiene. Shortly after eruption, most cracks in the occlusal surfaces of molar occlusal surfaces show signs of incipient caries [35]. The deep grooves and fissures, as well as the operculum covering the distal portion of the erupted FPM, allow bacterial biofilm to accumulate and be retained. The amount of bacterial biofilm accumulated at the occlusal surface was found to be higher in partially erupted teeth [40]. The results of our study are in agreement with those recorded in studies conducted by Carvalho J. and Manepalli S. They reported that caries localization at the occlusal level is the most frequent and has a very rapid evolution [41,42].

The predominant involvement of mandibular molars would be explained by their earlier eruption compared to their maxillary counterparts, therefore exposing them for a longer time to the oral cavity conditions and making them more prone to caries attacks [43,44]. Bassir et al., in their study, reached the same conclusions regarding the more frequent involvement of mandibular molars, claiming the differences in morphology and earlier eruption of mandibular molars as favorable factors [45].

## 5. Conclusions

The present study showing the prevalence of caries at the FPM level, in correlation with favoring and predisposing factors, provides the following results:

- Subjects belonging to the 13–19 age group have a higher prevalence of dental caries than the 6–12 age group. Future prevention programs in this age range should be given special attention.
- The gender of the subjects did not have a significant influence on the prevalence of dental caries.
- Irrespective of the parameters analyzed (age, environment, gender), the occlusal surface was the most affected surface, followed at a great distance by the other surfaces.
- At the same time, a much higher interest could be observed for the lower molars, both in terms of identified carious lesions and odontal restorations.
- The type of snacks influences the frequency of dental brushing, irrespective of age category, with sweet-type snacks attracting increased dental brushing.
- The highest percentage of children consume two snacks per day, and regardless of the frequency of these snacks, sweet snacks are found in the majority percentage.

## Figures and Tables

**Table 1:** ICDAS criteria.

Coding	Coding Description
0	healthy tooth
1	visible demineralization in enamel, only after prolonged drying
2	visible color changes in the enamel
3	lack of substance in enamel, without affecting dentin
4	pigmented dentin
5	visible cavity in the dentin
6	enlarged and visible cavity in the dentin

**Table 2:** Distribution of the study group with respect to gender, age group, and residence.

Gender	Total	6–12 Years Old—n (%)			13–19 Years Old—n (%)		
		Urban	Rural	Total	Urban	Rural	Total
Girls	171	77 (55.4%)	62 (44.6%)	139 (100%)	20 (62.5%)	12 (37.5%)	32 (100%)
Boys	140	58 (50.0%)	58 (50.0%)	116 (100%)	12 (50.0%)	12 (50.0%)	24 (100%)
Total		135	120	-	32	24	-
		255 children			56 children		
		311 children					

**Table 3:** Distribution of the study group by age groups divided by gender and residence.

Parameter		Age Group		Total	*p* *
		6–12 Years Old	13–19 Years Old		
Gender	Girls	139 (81.3%)	32 (18.7%)	171	0.720
	Boys	116 (82.9%)	24 (17.1%)	140	
Residence	Urban	135 (80.8%)	32 (19.2%)	167	0.568
	Rural	120 (83.3%)	24 (16.7%)	144	

* Chi-Square test.

**Table 4:** Study group distribution relative to FPM, odontal status, and age groups.

Status		Age Group		Total
		6–12 Years Old	13–19 Years Old	
M1.6	Indemn	217 (84.4%)	40 (15.6%)	257
	Absent	0 (0.0%)	1 (100%)	1
	Root remnant	2 (50.0%)	2 (50.0%)	4
	Untreated caries	33 (78.6%)	9 (21.4%)	42
	Partially treated	0 (0.0%)	0 (0.0%)	0
	Well treated	1 (50.0%)	1 (50.0%)	2
	Incorrectly treated	2 (40.0%)	3 (60.0%)	5
	*p*	0.007 *		-
M2.6	Indemn	223 (86.1%)	36 (13.9%)	259
	Absent	0 (0.0%)	1 (100%)	1
	Root remnant	0 (0.0%)	0 (0.0%)	0
	Untreated caries	28 (66.7%)	14 (33.3%)	42
	Partially treated	0 (0.0%)	0 (0.0%)	0
	Well treated	4 (66.7%)	2 (33.3%)	6
	Incorrectly treated	0 (0.0%)	3 (100%)	3
	*p*	<0.0005 *		-
M3.6	Indemn	177 (88.5%)	23 (11.5%)	200
	Absent	3 (75.0%)	1 (25.0%)	4
	Root remnant	3 (50.0%)	3 (50.0%)	6
	Untreated caries	59 (72.0%)	23 (28.0%)	82
	Partially treated	1 (100%)	0 (0.0%)	1
	Well treated	6 (66.7%)	3 (33.3%)	9
	Incorrectly treated	6 (66.7%)	3 (33.3%)	9
	*p*	0.005 *		-
M4.6	Indemn	173 (90.6%)	18 (9.4%)	191
	Absent	0 (0.0%)	1 (100%)	1
	Root remnant	4 (80.0%)	1 (20.0%)	5
	Untreated caries	67 (69.1%)	30 (30.9%)	97
	Partially treated	0 (0.0%)	0 (0.0%)	0
	Well treated	4 (80.0%)	1 (20.0%)	5
	Incorrectly treated	7 (58.3%)	5 (41.7%)	12
	*p*	<0.0005 *		-

* Chi-Square test.

**Table 5:** Distribution of the study group relative to FPM, gender, and residence.

Status		Age Group		Total	Residence	
		F	M		Urban	Rural
M1.6	Indemn	136 (52.9%)	121 (47.1%)	257	135 (52.5%)	122 (47.5%)
	Absent	1 (100%)	0 (0.0%)	1	1 (100%)	0 (0.0%)
	Root remnant	1 (25.0%)	3 (75.0%)	4	3 (75.0%)	1 (25.0%)
	Untreated caries	31 (73.8%)	11 (26.2%)	42	22 (52.4%)	20 (47.6%)
	Partially treated	0 (0.0%)	0 (0.0%)	0	0 (0.0%)	0 (0.0%)
	Well treated	1 (50.0%)	1 (50.0%)	2	2 (100%)	0 (0.0%)
	Incorrectly treated	1 (20.0%)	4 (80.0%)	5	4 (80.0%)	1 (20.0%)
	*p*	0.047 *		-	0.431 *	
M2.6	Indemn	138 (53.3%)	121 (46.7%)	259	139 (53.7%)	120 (46.3%)
	Absent	1 (100.0%)	0 (0.0%)	1	1 (100%)	0 (0.0%)
	Root remnant	0 (0.0%)	0 (0.0%)	0	0 (0.0%)	0 (0.0%)
	Untreated caries	28 (66.7%)	14 (33.3%)	42	20 (47.6%)	22 (52.4%)
	Partially treated	0 (0.0%)	0 (0.0%)	0	0 (0.0%)	0 (0.0%)
	Well treated	2 (33.3%)	4 (66.7%)	6	5 (83.3%)	1 (16.7%)
	Incorrectly treated	2 (66.7%)	1 (33.3%)	3	2 (66.7%)	1 (33.3%)
	*p*	0.315 *		-	0.432 *	
M3.6	Indemn	103 (51.5%)	97 (48.5%)	200	103 (51.5%)	97 (48.5%)
	Absent	2 (50.0%)	2 (50.0%)	4	2 (50.0%)	2 (50.0%)
	Root remnant	5 (83.3%)	1 (16.7%)	6	5 (83.3%)	1 (16.7%)
	Untreated caries	50 (61.0%)	32 (39.0%)	82	44 (53.7%)	38 (46.3%)
	Partially treated	0 (0.0%)	1 (100%)	1	1 (100%)	0 (0.0%)
	Well treated	6 (66.7%)	3 (33.3%)	9	8 (88.9%)	1 (11.1%)
	Incorrectly treated	5 (55.6%)	4 (44.4%)	9	4 (44.4%)	5 (55.6%)
	*p*	0.437 *		-	0.225 *	
M4.6	Indemn	102 (53.4%)	89 (46.6%)	191	96 (50.3%)	95 (49.7%)
	Absent	1 (100.0%)	0 (0.0%)	1	0 (0.0%)	1 (100%)
	Root remnant	4 (80.0%)	1 (20.0%)	5	3 (60.0%)	2 (40.0%)
	Untreated caries	56 (57.7%)	41 (42.3%)	97	56 (57.7%)	41 (42.3%)
	Partially treated	0 (0.0%)	0 (0.0%)	0	0 (0.0%)	0 (0.0%)
	Well treated	2 (40.0%)	3 (60.0%)	5	4 (80.0%)	1 (20.0%)
	Incorrectly treated	6 (50.0%)	6 (50.0%)	12	8 (66.7%)	4 (33.3%)
	*p*	0.678 *		-	0.418 *	

* Chi-Square test.

**Table 6:** ICDAS analysis (demographic data).

Tooth	Parameter	*p*				
		Occlusal	Mesial	Vestibular	Distal	Oral
M1.6	Age group	0.700	>0.05	>0.05	>0.05	>0.05
	Gender	0.046	>0.05	>0.05	>0.05	>0.05
	Environment	>0.05	>0.05	>0.05	>0.05	>0.05
M2.6	Age group	0.015	<0.0005	>0.05	>0.05	>0.05
	Gender	>0.05	>0.05	>0.05	0.022	>0.05
	Environment	>0.05	>0.05	>0.05	>0.05	>0.05
M3.6	Age group	0.015	>0.05	>0.05	>0.05	>0.05
	Gender	>0.05	>0.05	>0.05	>0.05	>0.05
	Environment	>0.05	>0.05	>0.05	>0.05	>0.05
M4.6	Age group	0.052	>0.05	>0.05	>0.05	>0.05
	Gender	>0.05	>0.05	>0.05	>0.05	>0.05
	Environment	>0.05	>0.05	>0.05	>0.05	>0.05

**Table 7:** Distribution of the study group with respect to the frequency of dental brushing.

Parameter		Dental Brushing Frequency				Total	*p* *
		2/day	1/day	1/3–4 days	1/7 days		
Age group (years old)	6–12	162 (63.5%)	63 (24.7%)	26 (10.2%)	4 (1.6%)	255	0.002
	13–19	21 (37.5%)	20 (35.7%)	12 (21.4%)	3 (5.4%)	56	
Gender	F	93 (54.4%)	48 (28.1%)	25 (14.6%)	5 (2.9%)	171	0.250
	M	90 (64.3%)	35 (25.0%)	13 (9.3%)	2 (1.4%)	140	
Residence	U	105 (62.9%)	43 (25.7%)	15 (9.0%)	4 (2.4%)	167	0.237
	R	78 (54.2%)	40 (27.8%)	23 (16.0%)	3 (2.1%)	144	

* Chi-Square test.

**Table 8:** Distribution of molar status relative to dental brushing frequency.

Status		Dental Brushing Frequency				Total	*p* *
		2/day	1/day	1/3–4 days	1/7 days		
M1.6	Indemn	176 (68.5%)	67 (26.1%)	14 (5.4%)	0 (0.0%)	257	<0.0005
	Absent	0 (0.0%)	0 (0.0%)	0 (0.0%)	1 (100.0%)	1	
	Root remnant	0 (0.0%)	0 (0.0%)	4 (100.0%)	0 (0.0%)	4	
	Untreated caries	5 (11.9%)	15 (35.7%)	16 (38.1%)	6 (14.3%)	42	
	Partially treated	0 (0.0%)	0 (0.0%)	0 (0.0%)	0 (0.0%)	0	
	Well treated	1 (50.0%)	1 (50.0%)	0 (0.0%)	0 (0.0%)	2	
	Incorrectly treated	1 (20.0%)	0 (0.0%)	4 (80.0%)	0 (0.0%)	5	
M2.6	Indemn	173 (66.8%)	70 (27.0%)	15 (5.8%)	1 (0.4%)	259	<0.0005
	Absent	0 (0.0%)	0 (0.0%)	0 (0.0%)	1 (100%)	1	
	Root remnant	0 (0.0%)	0 (0.0%)	0 (0.0%)	0 (0.0%)	0	
	Untreated caries	7 (16.7%)	9 (21.4%)	21 (50.0%)	5 (11.9%)	42	
	Partially treated	0 (0.0%)	0 (0.0%)	0 (0.0%)	0 (0.0%)	0	
	Well treated	2 (33.3%)	4 (66.7%)	0 (0.0%)	0 (0.0%)	6	
	Incorrectly treated	1 (33.3%)	0 (0.0%)	2 (66.7%)	0 (0.0%)	3	
M3.6	Indemn	158 (79.0%)	36 (18.0%)	5 (2.5%)	1 (0.5%)	200	<0.0005
	Absent	1 (25.0%)	2 (50.0%)	1 (25.0%)	0 (0.0%)	4	
	Root remnant	0 (0.0%)	0 (0.0%)	4 (66.7%)	2 (33.3%)	6	
	Untreated caries	17 (20.7%)	36 (43.9%)	25 (30.5%)	4 (4.9%)	82	
	Partially treated	0 (0.0%)	0 (0.0%)	1 (100%)	0 (0.0%)	1	
	Well treated	5 (55.6%)	4 (44.4%)	0 (0.0%)	0 (0.0%)	9	
	Incorrectly treated	2 (22.2%)	5 (55.6%)	2 (22.2%)	0 (0.0%)	9	
M4.6	Indemn	150 (78.5%)	37 (19.4%)	3 (1.6%)	1 (0.5%)	191	<0.0005
	Absent	0 (0.0%)	0 (0.0%)	1 (100%)	0 (0.0%)	1	
	Root remnant	1 (20.0%)	1 (20.0%)	1 (20.0%)	2 (40.0%)	5	
	Untreated caries	24 (24.7%)	41 (42.3%)	28 (28.9%)	4 (4.1%)	97	
	Partially treated	0 (0.0%)	0 (0.0%)	0 (0.0%)	0 (0.0%)	0	
	Well treated	3 (60.0%)	2 (40.0%)	0 (0.0%)	0 (0.0%)	5	
	Incorrectly treated	5 (41.7%)	2 (16.7%)	5 (41.7%)	0 (0.0%)	12	

* Chi-Square test.

**Table 9:** The type of snacks between meals.

Parameter		Type of Snacks				Total	*p* *
		Sweets	Salty Snacks	Fruit/Vegetables	Mixed		
Group	6–12	144 (56.5%)	20 (7.8%)	44 (17.3%)	47 (18.4%)	255	0.070
	13–19	38 (67.9%)	2 (3.6%)	3 (5.4%)	13 (23.2%)	56	
Gender	F	100 (58.5%)	10 (5.8%)	22 (12.9%)	39 (22.8%)	171	0.212
	M	82 (58.6%)	12 (8.6%)	25 (17.9%)	21 (15.0%)	140	
Residence	U	109 (65.3%)	9 (5.4%)	19 (11.4%)	30 (18.0%)	167	0.048
	R	73 (50.7%)	13 (9.0%)	28 (19.4%)	30 (20.8%)	144	
Snack frequency	3/day	20 (74.1%)	0 (0.0%)	5 (18.5%)	2 (7.4%)	27	0.0001
	2/day	58 (46.8%)	5 (4.0%)	31 (25.0%)	30 (24.2%)	124	
	1/day	46 (53.5%)	12 (14.0%)	10 (11.6%)	18 (20.9%)	86	
	Occasionally	44 (78.6%)	4 (7.1%)	1 (1.8%)	7 (12.5%)	56	
	Rarely/Never	14 (77.8%)	1 (5.6%)	0 (0.0%)	3 (16.7%)	18	
Dental brushing frequency	2/day	111 (60.7%)	16 (8.7%)	32 (17.5%)	24 (13.1%)	183	0.001
	1/day	49 (59.0%)	4 (4.8%)	14 (16.9%)	16 (19.3%)	83	
	1/3–4 days	19 (50.0%)	2 (5.3%)	1 (2.6%)	16 (42.1%)	38	
	1/7 days	3 (42.9%)	0 (0.0%)	0 (0.0%)	4 (57.1%)	7	

* Chi-Square test.

**Table 10:** Molar status in relation to the type of snacks.

Status		Type of Snacks				Total	*p* *
		Sweets	Salty Snacks	Fruit/Vegetables	Mixed		
M1.6	Indemn	148 (57.6%)	21 (8.2%)	47 (18.3%)	41 (16.0%)	257	0.043
	Absent	0 (0.0%)	0 (0.0%)	0 (0.0%)	1 (100.0%)	1	
	Root remnant	3 (75.0%)	0 (0.0%)	0 (0.0%)	1 (25.0%)	4	
	Untreated caries	26 (61.9%)	1 (2.4%)	0 (0.0%)	15 (35.7%)	42	
	Partially treated	0 (0.0%)	0 (0.0%)	0 (0.0%)	0 (0.0%)	0	
	Well treated	1 (50.0%)	0 (0.0%)	0 (0.0%)	1 (50.0%)	2	
	Incorrectly treated	4 (80.0%)	0 (0.0%)	0 (0.0%)	1 (20.0%)	5	
M2.6	Indemn	149 (57.5%)	22 (8.5%)	47 (18.1%)	41 (15.8%)	259	0.009
	Absent	0 (0.0%)	0 (0.0%)	0 (0.0%)	1 (100.0%)	1	
	Root remnant	0 (0.0%)	0 (0.0%)	0 (0.0%)	0 (0.0%)	0	
	Untreated caries	27 (64.3%)	0 (0.0%)	0 (0.0%)	15 (35.7%)	42	
	Partially treated	0 (0.0%)	0 (0.0%)	0 (0.0%)	0 (0.0%)	0	
	Well treated	4 (66.7%)	0 (0.0%)	0 (0.0%)	2 (33.3%)	6	
	Incorrectly treated	2 (66.7%)	0 (0.0%)	0 (0.0%)	1 (33.3%)	3	
M3.6	Indemn	107 (53.5%)	19 (9.5%)	45 (22.5%)	29 (14.5%)	200	0.002
	Absent	2 (50.0%)	0 (0.0%)	0 (0.0%)	2 (50.0%)	4	
	Root remnant	3 (50.0%)	1 (16.7%)	0 (0.0%)	2 (33.3%)	6	
	Untreated caries	57 (69.5%)	2 (2.4%)	2 (2.4%)	21 (25.6%)	82	
	Partially treated	1 (100.0%)	0 (0.0%)	0 (0.0%)	0 (0.0%)	1	
	Well treated	5 (55.6%)	0 (0.0%)	0 (0.0%)	4 (44.4%)	9	
	Incorrectly treated	7 (77.8%)	0 (0.0%)	0 (0.0%)	2 (22.2%)	9	
M4.6	Indemn	102 (53.4%)	18 (9.4%)	45 (23.6%)	26 (13.6%)	191	0.0001
	Absent	0 (0.0%)	0 (0.0%)	0 (0.0%)	1 (100%)	1	
	Root remnant	2 (40.0%)	0 (0.0%)	0 (0.0%)	3 (60.0%)	5	
	Untreated caries	64 (66.0%)	3 (3.1%)	2 (2.1%)	28 (28.9%)	97	
	Partially treated	0 (0.0%)	0 (0.0%)	0 (0.0%)	0 (0.0%)	0	
	Well treated	4 (80.0%)	0 (0.0%)	0 (0.0%)	1 (20.0%)	5	
	Incorrectly treated	10 (83.3%)	1 (8.3%)	0 (0.0%)	1 (8.3%)	12	

* Chi-Square test.

**Table 11:** ICDAS analysis.

Tooth	Parameter	*p*				
		Occlusal	Mesial	Vestibular	Distal	Oral
M1.6	Dental brushing	0.0001	<0.0005	<0.0005	0.0001	0.002
	Type of snacks	0.001	0.02	>0.05	>0.05	0.034
	Frequency of snacks	>0.05	>0.05	>0.05	>0.05	>0.05
M2.6	Dental brushing	0.0001	0.001	>0.05	<0.0005	0.001
	Type of snacks	0.002	>0.05	>0.05	>0.05	>0.05
	Frequency of snacks	0.005	0.007	>0.05	0.003	>0.05
M3.6	Dental brushing	<0.0005	0.015	0.0001	0.0001	>0.05
	Type of snacks	0.001	>0.05	0.038	>0.05	>0.05
	Frequency of snacks	0.009	>0.05	0.006	>0.05	>0.05
M4.6	Dental brushing	<0.0005	<0.0005	0.0001	0.005	>0.05
	Type of snacks	0.0001	0.021	0.005	0.021	>0.05
	Frequency of snacks	>0.05	>0.05	>0.05	>0.05	>0.05

**Table 12:** Type of snacks according to group age, gender, residence, and the frequency of dental brushing.

Parameter		Type of Snacks					Total	*p* *
		3/day	2/day	1/day	Occasionally	Never		
Group	6–12	18 (7.1%)	93 (36.5%)	75 (29.4%)	53 (20.8%)	16 (6.3%)	255	0.002
	13–19	9 (16.1%)	31 (55.4%)	11 (19.6%)	3 (5.4%)	2 (3.6%)	56	
Gender	F	15 (8.8%)	67 (39.2%)	50 (29.2%)	25 (14.6%)	14 (8.2%)	171	0.159
	M	12 (8.6%)	57 (40.7%)	36 (25.7%)	31 (22.1%)	4 (2.9%)	140	
Environment	U	19 (11.4%)	70 (41.9%)	42 (25.1%)	30 (18.0%)	6 (3.6%)	167	0.125
	R	8 (5.6%)	54 (37.5%)	44 (30.6%)	26 (18.1%)	12 (8.3%)	144	
Dental brushing frequency	2/day	17 (9.3%)	52 (28.4%)	61 (33.3%)	42 (23.0%)	11 (6.0%)	183	0.001
	1/day	8 (9.6%)	46 (55.4%)	18 (21.7%)	8 (9.6%)	3 (3.6%)	83	
	1/3–4 days	2 (5.3%)	21 (55.3%)	7 (18.4%)	4 (10.5%)	4 (10.5%)	38	
	1/7 days	0 (0%)	5 (71.4%)	0 (0%)	2 (28.6%)	0 (0%)	7	

* Chi-Square test.

**Table 13:** Molar status in relation to the frequency of snacks.

Status		Type of Snacks					Total	*p* *
		3/day	2/day	1/day	Occasionally	Never		
M1.6	Indemn	18 (7.0%)	91 (35.4%)	80 (31.1%)	52 (20.2%)	16 (6.2%)	257	0.078
	Absent	0 (0.0%)	1 (100.0%)	0 (0.0%)	0 (0.0%)	0 (0.0%)	1	
	Root remnant	1 (25.0%)	3 (75.0%)	0 (0.0%)	0 (0.0%)	0 (0.0%)	4	
	Untreated caries	6 (14.3%)	24 (57.1%)	6 (14.3%)	4 (9.5%)	2 (4.8%)	42	
	Partially treated	0 (0.0%)	0 (0.0%)	0 (0.0%)	0 (0.0%)	0 (0.0%)	0	
	Well treated	1 (50.0%)	1 (50.0%)	0 (0.0%)	0 (0.0%)	0 (0.0%)	2	
	Incorrectly treated	1 (20.0%)	4 (80.0%)	0 (0.0%)	0 (0.0%)	0 (0.0%)	5	
M2.6	Indemn	17 (6.6%)	92 (35.5%)	82 (31.7%)	51 (19.7%)	17 (6.6%)	259	0.010
	Absent	0 (0.0%)	1 (100.0%)	0 (0.0%)	0 (0.0%)	0 (0.0%)	1	
	Root remnant	0 (0.0%)	0 (0.0%)	0 (0.0%)	0 (0.0%)	0 (0.0%)	0	
	Untreated caries	8 (19.0%)	24 (57.1%)	4 (9.5%)	5 (11.9%)	1 (2.4%)	42	
	Partially treated	0 (0.0%)	0 (0.0%)	0 (0.0%)	0 (0.0%)	0 (0.0%)	0	
	Well treated	1 (16.7%)	5 (83.3%)	0 (0.0%)	0 (0.0%)	0 (0.0%)	6	
	Incorrectly treated	1 (33.3%)	2 (66.7%)	0 (0.0%)	0 (0.0%)	0 (0.0%)	3	
M3.6	Indemn	10 (5.0%)	59 (29.5%)	66 (33.0%)	49 (24.5%)	16 (8.0%)	200	0.0001
	Absent	0 (0.0%)	4 (100.0%)	0 (0.0%)	0 (0.0%)	0 (0.0%)	4	
	Root remnant	0 (0.0%)	5 (83.3%)	1 (16.7%)	0 (0.0%)	0 (0.0%)	6	
	Untreated caries	16 (19.5%)	44 (53.7%)	14 (17.1%)	6 (7.3%)	2 (2.4%)	82	
	Partially treated	0 (0.0%)	1 (100.0%)	0 (0.0%)	0 (0.0%)	0 (0.0%)	1	
	Well treated	1 (11.1%)	5 (55.6%)	3 (33.3%)	0 (0.0%)	0 (0.0%)	9	
	Incorrectly treated	0 (0.0%)	6 (66.7%)	2 (22.2%)	1 (11.1%)	0 (0.0%)	9	
M4.6	Indemn	11 (5.8%)	53 (27.7%)	66 (34.6%)	45 (23.6%)	16 (8.4%)	191	0.0001
	Absent	0 (0.0%)	1 (100%)	0 (0.0%)	0 (0.0%)	0 (0.0%)	1	
	Root remnant	0 (0.0%)	5 (100.0%)	0 (0.0%)	0 (0.0%)	0 (0.0%)	5	
	Untreated caries	14 (14.4%)	55 (56.7%)	16 (16.5%)	10 (10.3%)	2 (2.1%)	97	
	Partially treated	0 (0.0%)	0 (0.0%)	0 (0.0%)	0 (0.0%)	0 (0.0%)	0	
	Well treated	0 (0.0%)	2 (40.0%)	2 (40.0%)	1 (20.0%)	0 (0.0%)	5	
	Incorrectly treated	2 (16.7%)	8 (66.7%)	2 (16.7%)	0 (0.0%)	0 (0.0%)	12	

* Chi-Square test.

## Data Availability

The authors declare that the data from this research are available from the corresponding authors upon reasonable request.
